# There at Any Distance? Geographic Proximity and the Presence of Adult Children in Older People’s’ Confidant Networks

**DOI:** 10.1093/geroni/igaa057.1653

**Published:** 2020-12-16

**Authors:** Markus Schafer, Haosen Sun

**Affiliations:** University of Toronto, Toronto, Ontario, Canada

## Abstract

Adult children are key members of their aging parents’ close social network, often providing emotional and advisory supports. Still, adult children are not a guaranteed presence in older people’s core discussion networks. Geographical distance is a leading explanation for why some children are excluded from the confidant network, but we hypothesize that certain parent- and dyadic-level factors make these intergenerational ties more resilient to distance. Using wave six of the Survey of Health, Ageing, and Retirement in Europe, we identified whether a living adult child was also a member of the parent’s egocentric confidant network. We modeled the effect of the child (Level 1) and parent (Level 2) characteristics on the exclusion of a child from the core network using hierarchical logit models. We found that fifty-eight percent of children were excluded from a parent’s network. Parents were more likely to exclude those who lived more than 25 km compared to children who lived within 5 km. The impact of distance was exacerbated among parents who were older, partnered, or had four or more children. Parents with higher education and good computer skills were less sensitive to longer distances when listing a child as a confidant. Finally, parents who had confidants outside of the nuclear family and who lived in Northern Europe were less likely to exclude a child over 100 km from their confidant network. Together, results indicate that a number of demographic factors and personal and social resources contribute to the elasticity of parent-child ties across long distances.

